# Analysis of Migration and Adaptive Evolution in Tibetan Sheep Populations

**DOI:** 10.3390/ani16020317

**Published:** 2026-01-20

**Authors:** Wentao Zhang, Chao Yuan, Tingting Guo, Bowen Chen, Fan Wang, Jianbin Liu, Zengkui Lu

**Affiliations:** 1 Key Laboratory of Animal Genetics and Breeding on Tibetan Plateau, Ministry of Agriculture and Rural Affairs, Lanzhou Institute of Husbandry and Pharmaceutical Sciences, Chinese Academy of Agricultural Sciences, Lanzhou 730050, China; m18251871965@163.com (W.Z.); yuanchao@caas.cn (C.Y.); guotingting@caas.cn (T.G.); chenbowen@caas.cn (B.C.); 2 Sheep Breeding Engineering Technology Research Center, Chinese Academy of Agricultural Sciences, Lanzhou 730050, China; 3 College of Life Science and Engineering, Northwest Minzu University, Lanzhou 730030, China; wangfaneasy@126.com

**Keywords:** Tibetan sheep, whole-genome resequencing, selection signature, ultra-high altitude environment, cold and drought environment, hyper-arid environment

## Abstract

The genetic basis for Tibetan sheep adaptation to different high-altitude environments remains unknown. This study conducted whole-genome resequencing on 80 Tibetan sheep individuals from four major distribution areas on the Qinghai–Tibet Plateau. Based on the high-quality single-nucleotide polymorphisms (SNPs) obtained, an analysis of population-level genomic selection signals was performed. Population genomic analysis revealed that Tibetan sheep distributed across China originated in northern China but showed evidence of gene flow from South Asian sheep. Between populations from extremely high-altitude and mid-altitude regions, selection analyses identified five strongly positive selected genes (*HIF1AN* [Hypoxia Inducible Factor 1 Alpha Subunit Inhibitor], *HBE1* [Hemoglobin Subunit Epsilon 1], *HBE2* [Hemoglobin Subunit Epsilon 2], *TNFAIP3* [TNF Alpha Induced Protein 3], *RAD50* [RAD50 Double Strand Break Repair Protein]). These genes are associated with adaptation to hypoxia and intense UV radiation in high-altitude environments. Selection analyses between populations from extremely high-altitude and mid-altitude regions identified five strongly selected genes (*HIF1AN* [Hypoxia Inducible Factor 1 Alpha Subunit Inhibitor], *HBE1* [Hemoglobin Subunit Epsilon 1], *HBE2* [Hemoglobin Subunit Epsilon 2], *TNFAIP3* [TNF Alpha Induced Protein 3], *RAD50* [RAD50 Double Strand Break Repair Protein]) associated with hypoxia and intense UV radiation in high-altitude environments. Comparative genomic analyses of populations in cold and arid environments identified several candidate genes related to energy and water homeostasis, as well as hair development (*TP53* [Tumor Protein P53], *ATG101* [Autophagy Related 101], *ATP12A* [ATPase H+/K+ Transporting Non-Gastric Alpha2 Subunit], *KRT80* [Keratin 80], *KRT7* [Keratin 7]). Additionally, Tibetan sheep in the high-altitude arid deserts exhibit stronger adaptive selection for energy homeostasis and water utilization; meanwhile, the HIF-1 [Hypoxia Inducible Factor 1] signaling pathway helps counteract oxidative stress induced by extreme water scarcity in the plateau environment. Our study supports the hypothesis that Tibetan sheep originated in northern China and identifies distinct adaptive features in the Tibetan sheep genome corresponding to their habitats.

## Simple Summary

The environmental conditions on the Qinghai–Tibet Plateau vary widely, ranging from the warm and humid grassland climate at mid-altitudes in the northeast to the cold, arid, and desertified climate at high altitudes in the central and western regions. However, previous genomic studies on high-altitude adaptation have primarily focused on comparisons between mid-to-high altitudes and low altitudes, neglecting the complexity of conditions within the plateau itself. Investigating the mechanisms of adaptation to different high-altitude environments will enhance our understanding of how species adapt to the extreme conditions of the plateau.

## 1. Introduction

Ecologists are keenly interested in how organisms, especially those in extreme environments, adapt to challenging conditions. High-altitude regions (≥2500 m), covering 7% of Earth’s land area, present a combination of low oxygen, high radiation [1,2,3], temperature extremes, and aridity [4,5]. These conditions make them a natural laboratory for studying biological adaptation.

Genomic studies indicate that multiple high-altitude species, including humans, deer mice, and birds, exhibit significant convergent evolution at the genetic level in response to hypoxic environments. This is primarily reflected in enhanced oxygen transport capacity (e.g., through hemoglobin regulation) and optimized energy metabolism (e.g., via mitochondrial and thermogenesis-related genes) [6,7,8,9,10,11,12,13,14,15,16,17,18]. However, such research has largely focused on comparisons between high- and low-altitude populations, overlooking the diversity of climatic and geological conditions within high-altitude regions [19].

The Qinghai–Tibet Plateau, the world’s highest and largest plateau, features a harsh and heterogeneous climate [20]. Its ecological conditions deteriorate with rising altitude—shifting from relatively mild eastern regions to cold, arid, and desertified western highlands, accompanied by declining biodiversity [21]. Across most of its expanse, the environment is predominantly high-altitude, cold, and arid, with limited hospitable zones.

Tibetan sheep, historically associated with human ascent onto the plateau, have thrived under these severe conditions. Their remarkable resilience—surviving hypoxia, cold, and aridity—extends beyond behavior and physiology to their genomes [22]. Varied climatic pressures across altitudes have likely driven differential genomic evolution among Tibetan sheep populations, making them an ideal model for uncovering the genetic basis of mammalian adaptation to diverse high-altitude environments.

By analyzing genomic variations in the same species across diverse high-altitude environments, it is possible to more accurately elucidate the specific links between environmental selection and genetic adaptation. Therefore, this study selects 80 Tibetan sheep from the Qinghai–Tibet Plateau for whole-genome resequencing. By analyzing the population genetic structure and selective signals under specific environmental conditions, it aims to explore the genetic adaptation mechanisms of Tibetan sheep to various harsh climatic environments of the high-altitude region, including high elevation, cold, and aridity, in order to better understand their survival strategies under extreme conditions. This thesis will fill a scientific gap in understanding the adaptability of Tibetan sheep to the primary climatic environmental factors of the Qinghai–Tibet Plateau.

## 2. Materials and Methods

### 2.1. Ethics Statement

The experimental protocols involving sheep were approved by the Animal Ethics Committee at the Lanzhou Institute of Husbandry and Pharmaceutical Sciences, Chinese Academy of Agricultural Sciences (protocol code NO. 20231447 and 21 March 2023).

### 2.2. Sample Collection, Sequencing

To ensure the representativeness and comparability of the samples, in 2023, we selected 80 healthy and robust adult (2 years old) male sheep as the samples (20 Tao sheep, TS; 20 Awong sheep, AS; 20 Gamba sheep, GS; 20 Huoerba sheep, HS) (see Table S4). We chose half-siblings as the samples to minimize the differences among individuals. Venous blood samples (approximately 5 mL per animal) were aseptically collected from the jugular vein of each sheep using sterile vacuum tubes containing EDTA-K2 as an anticoagulant. Prior to collection, the puncture site was shaved and disinfected with 70% ethanol. DNA was extracted using the TIANamp Blood DNA Kit (Tian Gen Biotech Co. Ltd., Beijing, China), and its purity and concentration were measured using a Nanodrop 2000 (Thermo Scientific, Wilmington, NC, USA). The DNA was fragmented enzymatically, repaired to have flat ends, and dA tails were added. Sequencing adapters were attached, and the fragments were purified using AMPure XP beads (Beckman Coulter, Indianapolis, IN, USA). Fragments of 300–400 bp were selected for PCR amplification. The library was then purified, checked, and sequenced on Hiseq X10 PE150 (Illumina, San Diego, CA, USA). Raw reads were stored in FASTQ format for analysis.

### 2.3. Quality Control and Alignment

The filtering process [23] involved: (1) removing reads with adapters, (2) removing reads with over 10% N ratio, and (3) removing low-quality reads (more than 50% of bases with Q ≤ 20). High-quality reads were aligned to the Self-assembled genome_HB using BWA (0.7.15) [24] with MEN algorithm (parameters: -k 32 -M). S AM format results were converted to BAM using SAMtools (1.17). Duplicate reads were labeled with Picard (2.18.7). Coverage was counted with bedtools (v2.25.0) [25]. ANNOVAR [26] was used for variant annotation. SNPs were filtered by removing loci with over 20% missing rate and a minor allele frequency (MAF) of less than 5% [27,28].

### 2.4. Population Structure Analysis

To correct for Linkage Disequilibrium (LD), SNPs were trimmed using PLINK 1.09’s [29] indep-pairwise [30] function (parameters: 25 SNP window, 5 SNP step size, r2 threshold of 0.05). Principal component analysis (PCA) was performed with PLINK 1.09 to identify genetic clusters. A neighbor-joining tree [31] was constructed using treebest (1.9.2) [32] and visualized with ITOL (v7) [33] (https://itol.embl.de/upload.cgi, accessed on 15 July 2024). Population genetic structure [34] was analyzed using PLINK (1.09) and frappe [35] with k values from 2 to 4. We applied SMCpp (v1.15.3) [36], which can analyze effective population size history from multiple individuals and is more effective for short time scales. For both analyses, we used a neutral mutation rate of u = 2.5 × 10^−8^ and a generation time of 1.5 years. For SMC++, all samples were included with parameters: -p 0.5 -m 2.5 × 10^−8^ -w 100 -em 20 -sp cubic.

### 2.5. Selection Signal Analyses

This study employed three methods for selection analysis: Pairwise Fixation Index (F_ST_) [37], π ratio [38,39], and Tajima’s D [40]. The combined F_ST_ and π ratio analysis can provide comprehensive information on genetic dynamics, helping to reveal selection signals and evolutionary patterns in populations adapting to different climatic environments. Then, Tajima’s D can further evaluate whether the identified loci show signs of neutrality or selection. Analyses were conducted using PopGenome (2.7.5) [41,42] on filtered SNPs with sliding windows of 100 kb and steps of 10 kb [23]. Graphs were created using R (4.2.2) scripts [43].

### 2.6. Analysis of Candidate Genes

Next, we identified candidate selection signals for adaptation to different climates. We used the top 5% of F_ST_ and π ratios to filter overlapping SNP loci, which served as candidate signals. Tajima’s D was employed to assess SNP genetic differentiation. Gene annotation was performed using ANNOVAR.

Gene Ontology (GO) and Kyoto Encyclopedia of Genes and Genomes (KEGG) enrichment analyses were conducted to understand regulatory mechanisms related to climatic adaptation. DAVID 6.8 [44] (https://davidbioinformatics.nih.gov/, accessed 7 July 2024) categorized the candidate genes by function. Pathway enrichment was performed with Kobas 3.0 [45] (http://bioinfo.org/kobas/, accessed 7 July 2024). GO-enriched and KEGG-enriched plots were generated using Bioinformatics [46] (https://www.bioinformatics.com.cn/, accessed 10 July 2024). Gene interactions for shared genes were analyzed using STRING (https://cn.string-db.org/, accessed 17 July 2024).

## 3. Results

### 3.1. Whole-Genome Sequencing and Genetic Variation

**Figure 1:**
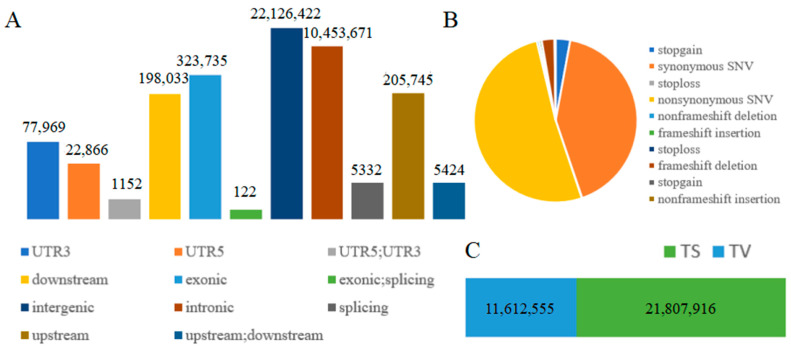
Types of Genetic Variations: (**A**) Positional Information; (**B**) Functional Information; (**C**) Base Substitution Types.

Whole-genome resequencing of 80 Tibetan sheep yielded 1.29 Tb of high-quality data (8,950,067,336 high-quality clean reads) after quality control (see Table S1; Table S2). The average total mapped rate was 99.29% (see Table S3), and the average sequencing depth was 6.3×. Variant annotation revealed a total of 33,420,471 SNPs. After filtering, 2,502,347 high-quality SNPs were identified. The TS/TV (Transition/Transversion) ratio was 1.9 (see Figure 1C), indicating a standardized genomic population structure. These SNPs were mainly distributed in downstream, exonic, intergenic, intronic, and upstream regions (see Figure 1A), suggesting that the high-altitude environment exerts selective pressure on the Tibetan sheep genome, concentrating on gene regulation, gene expression, and protein function changes. Analysis of SNP functional types (see Figure 1B) revealed a nonsynonymous to synonymous mutation ratio of 1.22, indicating overall positive selection or adaptive evolution of the genome [47]. These results provide a significant genetic basis for understanding how Tibetan sheep adapt to the harsh high-altitude environment.

### 3.2. Population Genetic Analysis

**Figure 2:**
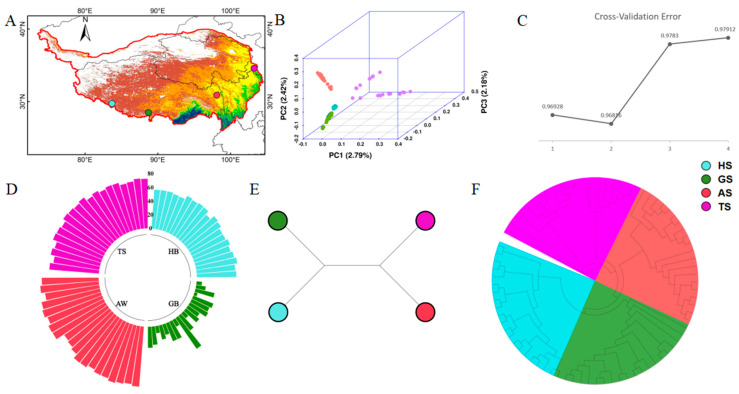
Geographic Distribution and Genetic Structure Analysis of Populations: (**A**) Geographic Distribution Map of Populations; (**B**) Principal Component Analysis; (**C**) Cross-Validation Error; (**D**) Genetic Component Analysis (K = 2); (**E**) Unrooted Evolutionary Tree of Populations; (**F**) Evolutionary Tree among Individuals.

The positions of the various Tibetan sheep breeds are shown in Figure 2A and Table S4. TS is located in the mid-altitude semi-humid region of northeastern Tibet, while AS (Awong Sheep), GS (Gamba Sheep), and HS (Huoerba Sheep) are distributed sequentially in the high-altitude semi-humid region of eastern Tibet, the high-altitude semi-arid region of southern Tibet, and the high-altitude arid region of southwestern Tibet. TS (Tao Sheep) and AS are situated in the temperate grassland climate, whereas GS and HS are in the subfrigid desertification climate. According to the PCA (principal component analysis) results (Figure 2B), TS and AS cluster together, whereas GS and HS show some overlap. Based on the cross-validation error (Figure 2C), K = 2 is identified as the optimal number of ancestral components. Subsequently, the genetic component analysis with K = 2 (Figure 2D) reveals that the proportion of the first ancestral component in TS, AS, and HS gradually decreases, while the intrusion of the second ancestral component in GS is apparent. According to the results of the unrooted population tree (Figure 2E) and the individual neighbor-joining tree (Figure 2E), the genetic relationship between TS and AS is closer, while the genetic relationship between GS and HS is closer. Population history analysis (see Figure S1) indicates that more than two thousand years ago, the populations had similar sizes and trends, suggesting that they shared the same genetic history. Additionally, all populations experienced a demographic bottleneck several thousand years ago, likely due to climate change. Over the past thousand years, population sizes have stabilized, and population structures have become fixed, with separation events completed. The TS population exhibited relatively small changes in size and an earlier growth, indicating that it might have been one of the earliest sheep populations to settle on the Qinghai–Tibet Plateau. The AS population showed a significant growth trend, occurring earlier than in other populations, suggesting that it was likely the next group to settle after TS. GS and HS were the last to settle, with GS only becoming fully stable in the past three hundred years. The Pi and F_ST_ indices of these populations (see Table S5; Table S6) also reflect that TS and AS are relatively isolated in their respective ecological environments, with limited gene flow. In contrast, there has been more gene flow between GS and HS, supporting the hypothesis of their close geographical and genetic connection.

### 3.3. Genetic Adaptation to High-Altitude Environment of Tibetan Sheep

**Figure 3:**
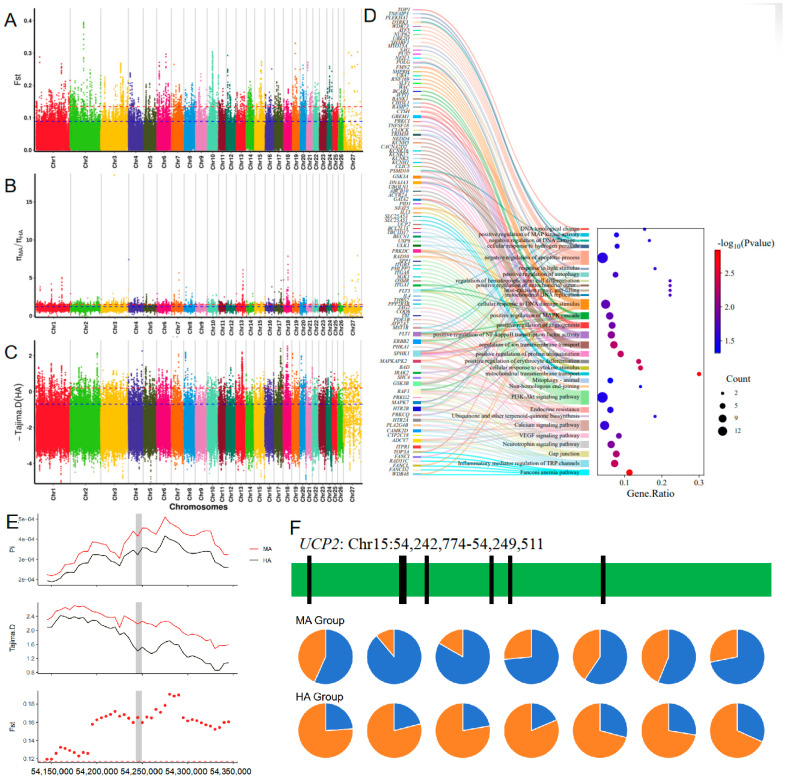
Regions of Tibetan sheep genome with strong selection signals at high altitude (Control group: MA): (**A**–**C**) Whole-genome scans by F_ST_, π ratio and Tajima’s D methods; (**D**) Results of functional enrichment of candidate genes; (**E**) Scanning of the 100 kb region above and below the *UCP2* gene; (**F**) Allele frequencies of the top seven mutation sites in the *UCP2* gene between high and low altitude populations.

The TS population (Medium Altitude group: MA) inhabits the mid-altitude regions of the Qinghai–Tibet Plateau, while the AS, GS, and HS populations (High-Altitude group: HA) reside in high-altitude areas. To identify potential candidate mutations that may have undergone positive selection in populations adapted to high-altitude environments, we employed three analysis methods to scan the genome regions (HA vs. MA) for signatures of positive selection. We identified 2577 significant SNP loci and annotated 684 candidate genes (see Figure 3A–C; Table S7). Functional analysis (see Figure 3D, Table 1) indicates that these candidate genes are associated with hypoxic adaptation and UV radiation resistance.

In the comparison between the HA and MA groups, *UCP2* exhibited a high F_ST_ and significant differences in Pi values (see Figure 3E), while the negative Tajima’s D value also suggested strong positive selection in this gene. Additionally, the mutation frequency at eight loci in the HA group was significantly lower than in the MA group (see Figure 3F), with four of these loci located in the 3′ UTR3, 5′ UTR, and exonic regions. These results indicate that the *UCP2* gene may have undergone positive selection in the high-altitude populations, and specific mutations in the 3′ UTR3, 5′ UTR, and exonic regions may have deleterious effects in high-altitude environments, leading to their selective elimination. This further supports the notion that these mutations do not confer an advantage for high-altitude hypoxic adaptation. Additionally, the following candidate genes (*HIF1AN*, *HBE1*, *HBE2*, *TNFAIP3*, *RAD50*, *NEIL1*) exhibited strong selective sweeps and significant mutation sites, which are associated with hypoxic adaptation or UV radiation resistance.

### 3.4. Genetic Adaptation to Cold Drought Environment of Tibetan Sheep

**Figure 4:**
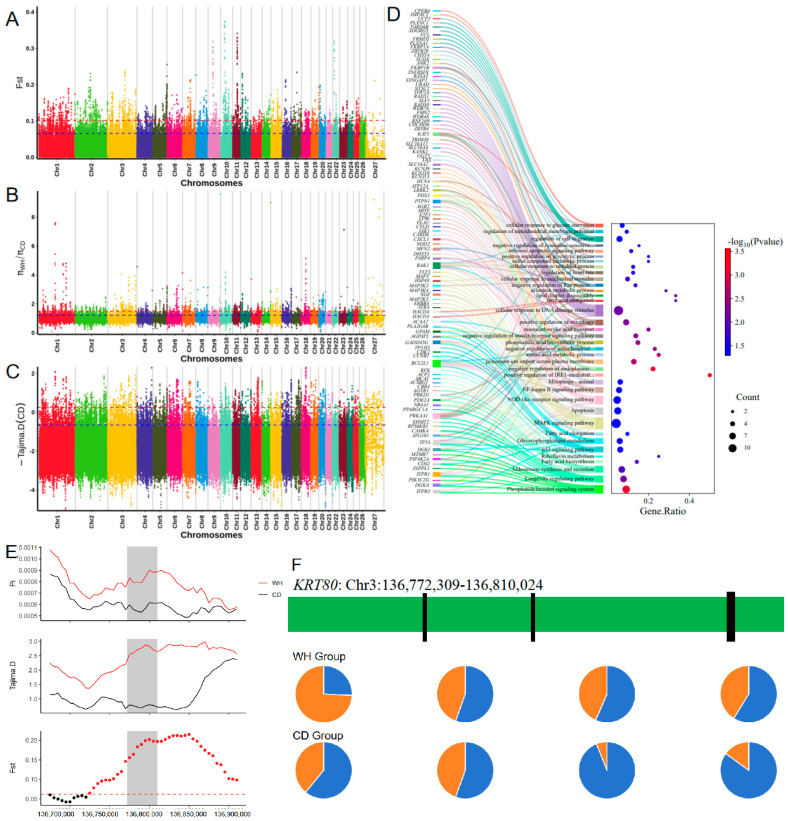
Regions of Tibetan sheep genome with strong selection signals in the cold drought environment of the plateau (Control group: WH): (**A**–**C**) Whole-genome scans by F_ST_, π ratio and Tajima’s D methods; (**D**) Results of functional enrichment of candidate genes; (**E**) Scanning of the 100 kb region above and below the *KRT80* gene; (**F**) Allele frequencies of the top four mutation sites in the *KRT80* gene between worm and cold altitude populations.

Considering the temperature and aridity differences in the habitats of Tibetan sheep, we compared the TS and AS populations, which reside in the temperate, humid regions of the plateau (Warm Humid group: WH), with the GS and HS populations, which live in the cold, arid desert regions of the southern and southwestern plateau (Cold Drought group: CD). To identify mutations that may contribute to adaptation to the cold and arid environments of the plateau, we used three analysis methods to scan for signatures of positive selection in genomic regions (CD vs. WH). We identified 2848 significant SNP loci and annotated 684 candidate genes (see Figure 4A–C; Table S8).

Functional analysis (see Figure 4D, Table 1) revealed that the significant biological processes (BPs) and pathways are mainly involved in protein folding and repair, DNA damage repair and cellular maintenance, mitochondrial function and energy metabolism, ion homeostasis, fatty acid metabolism and membrane fluidity, stress response, and antioxidative processes.

In the comparison between the CD and WH groups, the *KRT80* gene exhibited a very high F_ST_ and a relatively high Pi ratio (see Figure 4E), while the negative Tajima’s D value suggests strong selection in cold and arid environments. Additionally, the mutation frequency at four loci in the CD group was significantly higher than in the WH group (see Figure 4F), with two of these loci being nearly fixed (frequency close to 1) in the CD group. These results indicate that the *KRT80* gene may have undergone positive selection in populations adapted to cold and arid conditions. Although these mutations occur in intron regions and do not directly affect the amino acid sequence of the protein, they may potentially influence gene regulatory mechanisms, thereby affecting the gene’s role in the skin and hair follicles. Additionally, *KRT7*, *ATG101*, *ATP12A*, and *TP53* are also advantageous genes for animal adaptation to cold and arid environments.

### 3.5. Genetic Adaptation to Super Drought Plateau Environment of Tibetan Sheep

**Figure 5:**
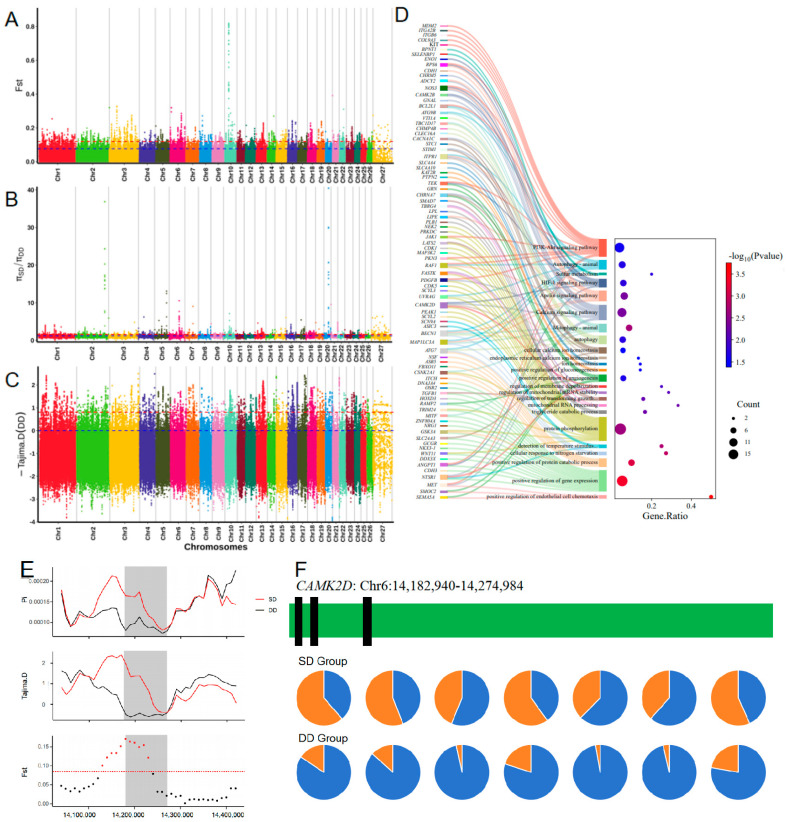
Regions of Tibetan sheep genome with strong selection signals in the super drought environment of the plateau (Control group: SD): (**A**–**C**) Whole-genome scans by F_ST_, π ratio and Tajima’s D methods; (**D**) Results of functional enrichment of candidate genes; (**E**) Scanning of the 100 kb region above and below the *CAMK2D* gene; (**F**) Allele frequencies of the top seven mutation sites in the *CAMK2D* gene between extreme arid semi-arid altitude populations.

To identify candidate mutations potentially under positive selection in populations from extremely arid environments on the plateau, we compared the HS population from southwestern Tibet (Drought Desert group: DD) with the GS population from southern Tibet (Subdrought Desert group: SD). A total of 2497 significant SNP loci were identified, and 608 candidate genes were annotated (see Figure 5A–C; Table S9).

The enrichment analysis results (see Figure 5D, Table 1) mainly involved ion homeostasis, angiogenesis and water utilization, stress response, energy metabolism, and antioxidative processes.

In the comparison between the DD and SD groups, the *CAMK2D* gene exhibited a very high F_ST_ and Pi ratio (see Figure 5E), and the negative Tajima’s D value suggests strong selection in the extremely arid desert environment of the plateau. Additionally, the mutation frequency at 14 loci in the DD group was significantly higher than in the SD group (see Figure 5F). This further indicates that these mutations may have a significant advantage in high-altitude adaptation. This suggests that the *CAMK2D* gene might be under positive selection in high-altitude populations. Although these mutations occur in intron regions, they may influence the maintenance of cellular water homeostasis by altering gene regulatory processes. Additionally, genes such as *PTPN2* and *SELENBP1* are also related to animal adaptation to extremely arid high-altitude environments.

## 4. Discussion

Based on whole-genome data, this study reveals the evolutionary origin of Tibetan sheep and their genetic adaptation mechanisms to the diverse extreme environments of the Qinghai–Tibet Plateau. Comprehensive analyses indicate that: first, Tibetan sheep primarily originated from sheep populations in northern China, and their population dynamics are closely linked to historical climatic events and trade routes (e.g., the Yadong Port); second, Tibetan sheep have adapted to different environmental pressures—such as hypoxia and intense radiation at ultra-high altitudes, cold aridity, and extreme drought—by selecting genes related to hypoxia adaptation, UV resistance, energy homeostasis, water balance, and hair development. The genetic basis of their origin and adaptation to these varied environments is discussed in detail below.

### 4.1. Genomic Evidence Supports Northern China Origin Hypothesis

Although the Holocene climate was relatively warm and humid, it was punctuated by multiple cold and arid events. This led to habitat degradation and reduced food resources, which may have impacted the survival of Tibetan sheep and caused a decline in their population numbers. Additionally, climate change led to alterations in vegetation structure, reducing the area of grasslands and pastures, which further diminished food resources. As the ecological environment changed, competition and predation pressures from other animal species also increased, negatively impacting the survival of Tibetan sheep. After this population bottleneck, the TS and AS populations were able to rapidly grow one after the other. It is largely attributed to the relatively favorable environments in their respective habitats. The GS population, located in Gamba County, is adjacent to the Yadong port. It was one of the most active trade and exchange hubs with South Asia along the ancient Tea Horse Road. Thus, we speculate that the long-term genetic exchange in this area significantly increased the presence of a second ancestral component in the GS population. SMCpp analysis indicates that the GS population only began to stabilize about 300 years ago; This coincides with the period when trade at Yadong port was most frequent in the mid-17th century. This temporal correlation further supports the hypothesis that the frequent exchanges at Yadong port had a significant impact on the genetic structure of the GS population.

It is noteworthy that the inferred migration route of Tibetan sheep based on genomic demographic history closely resembles the Tang-Bo Ancient Road [48]. Specifically, the inferred route of Tibetan sheep spreading to the interior via the northeastern fringe region is highly consistent with archeological evidence and ancient Chinese records of human history [48,49]. Furthermore, the genomic analysis of the GS sheep population does not support the hypothesis of an origin in the South Asian subcontinent. This suggests that, despite gene flow from South Asia, the South Asian subcontinent was not the primary origin of Tibetan sheep. Instead, South Asian sheep may have only exerted a genetic influence on certain specific Tibetan sheep populations, such as the GS population, rather than serving as the main genetic source of Tibetan sheep. Therefore, although there was an influx of South Asian subcontinent sheep into southern Tibet, this does not alter the overall conclusion that Tibetan sheep primarily originated from sheep populations in northern China [50,51,52].

### 4.2. Adaptation of Tibetan Sheep to the Hypoxic and Intense Ultraviolet Environment at Ultra-High Altitudes

Humans and other organisms are relatively well-adapted to mid-altitude regions, but their adaptability to extremely high-altitude environments is poor. The primary environmental stressors at high altitudes are low oxygen levels and intense ultraviolet (UV) radiation. In extremely high-altitude areas, the oxygen content is only 50% of that at sea level. It has reached the physiological limits for organisms and can lead to hypoxia and energy metabolism disorders. To survive in the hypoxic conditions of extremely high altitudes, Tibetan sheep adjust their hypoxia adaptation through three mechanisms: oxygen transport, energy utilization, and hypoxia protection. Firstly, they enhance oxygen transport capacity by promoting angiogenesis and increasing erythropoiesis. Secondly, they maintain energy metabolism in hypoxic environments by enhancing mitochondrial function. Additionally, they protect cells in hypoxic conditions by maintaining ion homeostasis and activating various response mechanisms. *HIF1AN* regulates the expression of hypoxia-responsive genes by modulating the stability and activity of HIF1α [53,54]. *UCP2* aids in adapting to hypoxic conditions by regulating energy metabolism and reducing oxidative stress [55,56]. *HBE1* and *HBE2* improve oxygen acquisition and transport efficiency by increasing hemoglobin’s oxygen-carrying capacity [57,58]. These genes work together to help the body maintain normal metabolic function and oxygen supply under hypoxic conditions, thereby enhancing hypoxia adaptation.

In high-altitude regions, ultraviolet exposure increases by approximately 10% with every 1000 m rise in altitude [59,60]. Intense UV radiation has sufficient energy to penetrate cells and cause the formation of covalent bonds between bases in DNA strands (especially thymine), leading to mutations [61]. It can also stimulate the production of reactive oxygen species (ROS) within the body, triggering oxidative stress and damaging key molecules within cells. Additionally, UV radiation can directly absorb amino acids in proteins, particularly tryptophan, tyrosine, and phenylalanine. This absorption can cause the covalent bonds within protein molecules to break or alter the protein’s structure, leading to instability in the three-dimensional structure and subsequent denaturation. To counteract the damage caused by intense UV radiation at extremely high altitudes, Tibetan sheep may enhance their tolerance to UV radiation and survival by activating DNA damage repair, oxidative stress response, and cellular protection, as well as protein repair and degradation mechanisms. UV radiation can induce single-strand breaks and oxidative damage, and may even cause double-strand DNA breaks. *NEIL1* can recognize and excise these oxidized bases, initiating the BER repair process [62] to fix oxidative damage caused by UV radiation. RAD50, together with MRE11 and NBS1, forms the MRN complex [63], which recognizes and binds to double-strand break sites, promotes end processing, and initiates the repair process. The NF-κB signaling pathway [64] is activated in response to stress such as UV radiation, leading to the expression of genes related to inflammation and cell survival; *TNFAIP3* protects cells from UV radiation-induced cell death by inhibiting NF-κB activity [65]. The synergistic action of these genes enables cells to effectively respond to various forms of damage caused by UV radiation, enhancing cellular tolerance to intense UV exposure. These findings provide further evidence of the adaptation of Tibetan sheep to the hypoxic and intense UV radiation environment at extremely high altitudes.

### 4.3. Adaptation of Tibetan Sheep to Cold Drought Environment on the Plateau

The climate varies significantly across different regions of the Qinghai–Tibet Plateau, ranging from humid, temperate alpine grasslands and forest climates to arid, cold desert climates. The high-altitude cold and arid desert environment presents challenges such as cold temperatures, large temperature fluctuations, food shortages, and water scarcity. Our research findings indicate that Tibetan sheep in cold and arid environments exhibit significant positive selection in genes related to energy, structural repair, and ion homeostasis. DNA damage repair and protein folding repair are crucial mechanisms for maintaining genomic stability and protein function in cold and arid environments. The “Cellular response to glucose starvation” suggests that food scarcity is a concern in these harsh conditions. To cope with food shortages, fatty acid metabolism aids in energy storage. Additionally, improvements in mitochondrial function and energy metabolism help organisms meet increased energy demands and unstable energy supply in cold and arid environments. The regulation of fatty acid metabolism and membrane structure/function enhances the resilience and stability of cell membranes to survive harsh conditions. Ion homeostasis mechanisms also maintain stable ion concentrations in arid environments, preventing dehydration and electrolyte imbalances. Aridity led to the accumulation of reactive oxygen species (ROS), and antioxidant mechanisms work to neutralize excessive ROS, reducing oxidative stress and protecting cellular structure and function.

*TP53* regulates the expression of heat shock proteins and other stress response-related genes, controlling the cell cycle and apoptosis to enhance stress resistance [66,67]. *ATG101* maintains cellular homeostasis and survival through autophagy [68,69]. *ATP12A* is thought to help maintain ion balance in the kidneys and epithelial tissues, preventing dehydration and osmotic imbalance. *KRT80* and *KRT7*, members of the keratin family, are involved in forming and maintaining the skin barrier. Furthermore, we observed that Tibetan sheep in cold and arid regions have higher wool yields. This suggests that in cold and arid environments, mutations in the *KRT80* and *KRT7* genes might alter the structure and function of keratin, promoting wool growth and thickening to combat low temperatures and reduce heat loss from the body. The combined actions of these genes enable the organism to maintain normal physiological functions and survival capabilities in extreme cold or arid environments. These findings reveal the ability of Tibetan sheep to adapt to the harsh climatic conditions of the high-altitude cold and arid desert environments.

### 4.4. Adaptation of Tibetan Sheep to Extreme Arid Environment on the Plateau

The high-altitude arid desert climate is more extreme than semi-arid climates, posing a greater challenge to the organism’s water management and nutrient utilization efficiency. In arid regions, Tibetan sheep exhibit positive selection for genes related to ion homeostasis, angiogenesis, and water utilization. These help maintain fluid balance and improve water use efficiency. Additionally, to cope with food scarcity, the body mobilizes stored energy and regulates multiple energy metabolism mechanisms to sustain physiological functions and life activities. In the more arid, extreme high-altitude environment, hypoxia is more severe. On one hand, to maintain core body temperature, the body constricts peripheral blood vessels. On the other hand, dehydration further leads to blood concentration and reduced blood volume. *CAMK2D* and *PTPN2* help maintain cellular water and electrolyte balance in arid environments by regulating calcium signaling and kidney function, respectively [70,71]. The HS population not only enhances angiogenesis and maintains vascular structure but also activates the HIF-1 signaling pathway to resist severe hypoxic stress. *SELENBP1* plays a key role in antioxidant defense [72], protecting cells from oxidative damage caused by reactive oxygen species (ROS) in arid and hypoxic conditions, thus maintaining cell survival. However, the HIF-1 signaling pathway was not enriched in the comparison between high and medium altitudes. This might be because the difference in oxygen partial pressure between the two altitudes is not significant enough, and high-altitude sheep may have adapted to a relatively lower oxygen supply through other non-HIF-1 dependent mechanisms, such as promoting angiogenesis and improving oxygen transport. These findings further reveal the specific adaptive capabilities of Tibetan sheep under extreme arid conditions.

## 5. Conclusions

The population genomic analysis of Tibetan sheep supports a northern Chinese origin. However, there is still a lack of evidence to support the southern origin hypothesis. Comparative genomics reveals a genetic basis for adaptation to the plateau’s diverse extreme environments: *HIF1AN*, *HBE1*, *HBE2*, and *UCP2* are linked to high-altitude hypoxia; *NEIL1*, *TNFAIP3*, and *RAD50* to UV resistance; and energy/water homeostasis genes to cold and aridity. Under oxidative stress induced by water scarcity, the HIF-1 pathway and *SELENBP1* likely contribute to supporting cellular energy metabolism and survival. These findings deepen our understanding of Tibetan sheep’s adaptive evolution and provide insights into their evolutionary origins and survival strategies in extreme environments.

## Figures and Tables

**Table 1:** Major Biological Functions and Pathways Associated with Candidate Genes.

Adaptation Focus	Functional Category	Associated Biological Processes/Pathways
Hypoxic Adaptation	Enhanced Oxygen Delivery	Positive regulation of erythrocyte differentiation; Regulation of hematopoietic stem cell differentiation
	Mitochondrial & Energy Regulation	Mitochondrial transmembrane transport; Mitophagy-animal
	Cellular Protection under Hypoxia	Negative regulation of apoptotic process; Positive regulation of MAPK cascade; PI3K-Akt signaling pathway
	Promotion of Angiogenesis	Positive regulation of angiogenesis; VEGF signaling pathway
	Ion Homeostasis	Regulation of ion transmembrane transport; Calcium signaling pathway
	Hypoxic Response Regulation	Cellular response to cytokine stimulus; Gap junction
UV Radiation Resistance	DNA Damage Repair & Maintenance	Cellular response to DNA damage stimulus; Base-excision repair, gap-filling
	Oxidative Stress Response & Cellular Protection	Cellular response to hydrogen peroxide; Positive regulation of autophagy
	Protein Repair & Degradation	Positive regulation of protein ubiquitination; Positive regulation of MAP kinase activity
	Radiation Response	Response to light stimulus
Genetic Adaptation to Cold & Arid Environment	Protein Folding & Repair	Cellular response to unfolded protein; Negative regulation of endoplasmic reticulum stress-induced intrinsic apoptotic signaling pathway
	DNA Damage Repair & Cellular Maintenance	Cellular response to DNA damage stimulus; Apoptosis
	Mitochondrial Function & Energy Metabolism	Regulation of mitochondrial membrane potential; Positive regulation of glycolytic process
	Ion Homeostasis	Potassium ion import across plasma membrane; Phosphatidylinositol signaling system
	Fatty Acid Metabolism & Membrane Fluidity	Fatty acid biosynthesis; Fatty acid elongation; Glycerophospholipid Metabolism
	Stress Response	MAPK signaling pathway; NF-kappa B signaling pathway; Positive Regulation of Autophagy
	Antioxidative Processes	Allantoin Metabolic Process; Riboflavin Metabolism
Genetic Adaptation to Hyper-Arid Plateau Environment	Ion Homeostasis	Regulation of membrane depolarization; Ion homeostasis
	Angiogenesis & Water Utilization	Positive regulation of endothelial cell chemotaxis; Positive regulation of angiogenesis; Apelin signaling pathway; Cellular response to nitrogen starvation
	Stress Response	HIF-1 signaling pathway; PI3K-Akt signaling pathway
	Energy Metabolism	Positive regulation of gluconeogenesis
	Antioxidative Processes	Sulfur metabolism

## Data Availability

Data are available upon request due to privacy/ethical restrictions.

## Supplementary Materials

The following supporting information can be downloaded at https://www.mdpi.com/article/10.3390/ani16020317/s1, Table S1: Statistics of base information before and after filtering; Table S2: Reads filter information statistics; Table S3: Total Mapped Rate; Table S4: Information of the sheep populations in this study; Table S5: F_ST_ index between groups; Table S6: Pi index in every population; Table S7: Candidate loci and genes for high altitude environment (HA vs. MA); Table S8: Candidate loci and genes for cold drought environment (CD vs. WH); Table S9: Candidate loci and genes for super drought environment (DD vs. SD); Table S10: GO enrichment of Candidate genes for high altitude environment (HA vs. MA); Table S11: KEGG enrichment of Candidate genes for high altitude environment (HA vs. MA); Table S12: GO enrichment of Candidate genes for cold drought environment (CD vs. WH); Table S13: KEGG enrichment of Candidate genes for cold drought environment (CD vs. WH); Table S14: GO enrichment of Candidate genes for super drought environment (DD vs. SD); Table S15: KEGG enrichment of Candidate genes for super drought environment (DD vs. SD); Figure S1: SMCpp of four groups of analysis.

## References

[1] Lillywhite H.B., Navas C.A. (2006). Animals, energy, and water in extreme environments: Perspectives from Ithala 2004. Physiol. Biochem. Zool..

[2] Swenson E.R., Bärtsch P. (2014). High Altitude.

[3] Kappen L. (1973). Response to extreme environments. The Lichens.

[4] Hillel D., Rosenzweig C. (2002). Desertification in relation to climate variability and change. Adv. Agron..

[5] Ward D. (2016). The Biology of Deserts.

[6] Cheviron Z., Brumfield R. (2012). Genomic insights into adaptation to high-altitude environments. Heredity.

[7] Beall C.M. (2014). Adaptation to high altitude: Phenotypes and genotypes. Annu. Rev. Anthropol..

[8] Bigham A.W., Wilson M.J., Julian C.G., Kiyamu M., Vargas E., Leon-Velarde F., Rivera-Chira M., Rodriquez C., Browne V.A., Parra E. (2013). Andean and tibetan patterns of adaptation to high altitude. Am. J. Hum. Biol..

[9] Beall C.M. (2006). Andean, tibetan, and ethiopian patterns of adaptation to high-altitude hypoxia. Integr. Comp. Biol..

[10] Weber R.E. (2007). High-altitude adaptations in vertebrate hemoglobins. Respir. Physiol. Neurobiol..

[11] Storz J.F., Sabatino S.J., Hoffmann F.G., Gering E.J., Moriyama H., Ferrand N., Monteiro B., Nachman M.W. (2007). The molecular basis of high-altitude adaptation in deer mice. PLoS Genet..

[12] Lutz P.L. (1980). On the oxygen affinity of bird blood. Am. Zool..

[13] Hassanin A., Ropiquet A., Couloux A., Cruaud C. (2009). Evolution of the mitochondrial genome in mammals living at high altitude: New insights from a study of the tribe caprini (bovidae, antilopinae). J. Mol. Evol..

[14] Di Rocco F., Parisi G., Zambelli A., Vida-Rioja L. (2006). Rapid evolution of cytochrome c oxidase subunit ii in camelids (tylopoda, camelidae). J. Bioenerg. Biomembr..

[15] Xu S., Luosang J., Hua S., He J., Ciren A., Wang W., Tong X., Liang Y., Wang J., Zheng X. (2007). High altitude adaptation and phylogenetic analysis of tibetan horse based on the mitochondrial genome. J. Genet. Genom..

[16] Fontanillas P., Dépraz A., Giorgi M.S., Perrin N. (2005). Nonshivering thermogenesis capacity associated to mitochondrial DNA haplotypes and gender in the greater white-toothed shrew, crocidura russula. Mol. Ecol..

[17] Luo Y., Gao W., Gao Y., Tang S., Huang Q., Tan X., Chen J., Huang T. (2008). Mitochondrial genome analysis of ochotona curzoniae and implication of cytochrome c oxidase in hypoxic adaptation. Mitochondrion.

[18] Cheviron Z.A., Brumfield R.T. (2009). Migration-selection balance and local adaptation of mitochondrial haplotypes in rufous-collared sparrows (zonotrichia capensis) along an elevational gradient. Evolution.

[19] Inouye D.W., Wielgolaski F.E. (2003). High altitude climates. Phenology: An Integrative Environmental.

[20] Liu X., Sun H., Miao Y., Dong B., Yin Z.-Y. (2015). Impacts of uplift of northern tibetan plateau and formation of asian inland deserts on regional climate and environment. Quat. Sci. Rev..

[21] Sun H., Niu Y., CHEN Y.S., Song B., LIU C.Q., PENG D.L., CHEN J.G., Yang Y. (2014). Survival and reproduction of plant species in the qinghai–tibet plateau. J. Syst. Evol..

[22] Hu X.-J., Yang J., Xie X.-L., Lv F.-H., Cao Y.-H., Li W.-R., Liu M.-J., Wang Y.-T., Li J.-Q., Liu Y.-G. (2019). The genome landscape of tibetan sheep reveals adaptive introgression from argali and the history of early human settlements on the qinghai–tibetan plateau. Mol. Biol. Evol..

[23] Zhang W., Jin M., Li T., Lu Z., Wang H., Yuan Z., Wei C. (2023). Whole-genome resequencing reveals selection signal related to sheep wool fineness. Animals.

[24] Li H., Durbin R. (2009). Fast and accurate short read alignment with burrows–wheeler transform. Bioinformatics.

[25] Quinlan A.R., Hall I.M. (2010). Bedtools: A flexible suite of utilities for comparing genomic features. Bioinformatics.

[26] Wang K., Li M., Hakonarson H. (2010). Annovar: Functional annotation of genetic variants from high-throughput sequencing data. Nucleic Acids Res..

[27] Zhang W., Jin M., Lu Z., Li T., Wang H., Yuan Z., Wei C. (2023). Whole genome resequencing reveals selection signals related to wool color in sheep. Animals.

[28] Zhang W., Luosang C., Yuan C., Guo T., Wei C., Liu J., Lu Z. (2024). Selection signatures of wool color in gangba sheep revealed by genome-wide snp discovery. BMC Genom..

[29] Purcell S., Neale B., Todd-Brown K., Thomas L., Ferreira M.A.R., Bender D., Maller J., Sklar P., de Bakker P.I.W., Daly M.J. (2007). Plink: A tool set for whole-genome association and population-based linkage analyses. Am. J. Hum. Genet..

[30] Wei C., Wang H., Liu G., Zhao F., Kijas J.W., Ma Y., Lu J., Zhang L., Cao J., Wu M. (2016). Genome-wide analysis reveals adaptation to high altitudes in tibetan sheep. Sci. Rep..

[31] Bruno W.J., Socci N.D., Halpern A.L. (2000). Weighted neighbor joining: A likelihood-based approach to distance-based phylogeny reconstruction. Mol. Biol. Evol..

[32] Vilella A.J., Severin J., Ureta-Vidal A., Heng L., Durbin R., Birney E. (2009). Ensemblcompara genetrees: Complete, duplication-aware phylogenetic trees in vertebrates. Genome Res..

[33] Letunic I., Bork P. (2019). Interactive tree of life (itol) v4: Recent updates and new developments. Nucleic Acids Res..

[34] Tang H., Quertermous T., Rodriguez B., Kardia S.L., Zhu X., Brown A., Pankow J.S., Province M.A., Hunt S.C., Boerwinkle E. (2005). Genetic structure, self-identified race/ethnicity, and confounding in case-control association studies. Am. J. Hum. Genet..

[35] Alexander D.H., Novembre J., Lange K. (2009). Fast model-based estimation of ancestry in unrelated individuals. Genome Res..

[36] Terhorst J., Kamm J.A., Song Y.S. (2017). Robust and scalable inference of population history from hundreds of unphased whole genomes. Nat. Genet..

[37] Weir B.S., Cockerham C.C. (1984). Estimating f-statistics for the analysis of population structure. Evolution.

[38] Nei M., Li W.-H. (1979). Mathematical model for studying genetic variation in terms of restriction endonucleases. Proc. Natl. Acad. Sci. USA.

[39] Lin T., Zhu G., Zhang J., Xu X., Yu Q., Zheng Z., Zhang Z., Lun Y., Li S., Wang X. (2014). Genomic analyses provide insights into the history of tomato breeding. Nat. Genet..

[40] Tajima F. (1989). Statistical method for testing the neutral mutation hypothesis by DNA polymorphism. Genetics.

[41] Pfeifer B., Wittelsbürger U., Ramos-Onsins S.E., Lercher M.J. (2014). Popgenome: An efficient swiss army knife for population genomic analyses in r. Mol. Biol. Evol..

[42] Gallone B., Steensels J., Prahl T., Soriaga L., Saels V., Herrera-Malaver B., Merlevede A., Roncoroni M., Voordeckers K., Miraglia L. (2016). Domestication and divergence of saccharomyces cerevisiae beer yeasts. Cell.

[43] R Core Team (2013). R: A Language and Environment for Statistical Computing.

[44] Huang D.W., Sherman B.T., Lempicki R.A. (2009). Systematic and integrative analysis of large gene lists using david bioinformatics resources. Nat. Protoc..

[45] Bu D., Luo H., Huo P., Wang Z., Zhang S., He Z., Wu Y., Zhao L., Liu J., Guo J. (2021). Kobas-i: Intelligent prioritization and exploratory visualization of biological functions for gene enrichment analysis. Nucleic Acids Res..

[46] Tang D., Chen M., Huang X., Zhang G., Zeng L., Zhang G., Wu S., Wang Y. (2023). Srplot: A free online platform for data visualization and graphing. PLoS ONE.

[47] Barreiro L.B., Laval G., Quach H., Patin E., Quintana-Murci L. (2008). Natural selection has driven population differentiation in modern humans. Nat. Genet..

[48] Zhang D., Dong G., Wang H., Ren X., Ha P.u., Qiang M., Chen F. (2016). History and possible mechanisms of prehistoric human migration to the tibetan plateau. Sci. China Earth Sci..

[49] Chen F.H., Dong G.H., Zhang D.J., Liu X.Y., Jia X., An C.-B., Ma M.M., Xie Y.W., Barton L., Ren X. (2015). Agriculture facilitated permanent human occupation of the tibetan plateau after 3600 bp. Science.

[50] Yang J., Li W.-R., Lv F.-H., He S.-G., Tian S.-L., Peng W.-F., Sun Y.-W., Zhao Y.-X., Tu X.-L., Zhang M. (2016). Whole-genome sequencing of native sheep provides insights into rapid adaptations to extreme environments. Mol. Biol. Evol..

[51] Lv F.-H., Peng W.-F., Yang J., Zhao Y.-X., Li W.-R., Liu M.-J., Ma Y.-H., Zhao Q.-J., Yang G.-L., Wang F. (2015). Mitogenomic meta-analysis identifies two phases of migration in the history of eastern eurasian sheep. Mol. Biol. Evol..

[52] Zhao Y.-X., Yang J., Lv F.-H., Hu X.-J., Xie X.-L., Zhang M., Li W.-R., Liu M.-J., Wang Y.-T., Li J.-Q. (2017). Genomic reconstruction of the history of native sheep reveals the peopling patterns of nomads and the expansion of early pastoralism in east asia. Mol. Biol. Evol..

[53] Berra E., Benizri E., Ginouvès A., Volmat V., Roux D., Pouysségur J. (2003). Hif prolyl-hydroxylase 2 is the key oxygen sensor setting low steady-state levels of hif-1alpha in normoxia. EMBO J..

[54] Epstein A.C., Gleadle J.M., McNeill L.A., Hewitson K.S., O’Rourke J., Mole D.R., Mukherji M., Metzen E., Wilson M.I., Dhanda A. (2001). C. Elegans egl-9 and mammalian homologs define a family of dioxygenases that regulate hif by prolyl hydroxylation. Cell.

[55] Echtay K.S., Roussel D., St-Pierre J., Jekabsons M.B., Cadenas S., Stuart J.A., Harper J.A., Roebuck S.J., Morrison A., Pickering S. (2002). Superoxide activates mitochondrial uncoupling proteins. Nature.

[56] Sluse F.E. (2012). Uncoupling proteins: Molecular, functional, regulatory, physiological and pathological aspects. Adv. Exp. Med. Biol..

[57] Yu H.-C., Cui R., Chen M.-Y., Du X.-Y., Bai Q.-R., Zhang S.-L., Guo J.-J., Tong F.-C., Wu J. (2025). Regulation of erythroid differentiation via the hif1α-nfil3-pim1 signaling axis under hypoxia. Antioxid. Redox Signal..

[58] Ginder G.D. (2015). Epigenetic regulation of fetal globin gene expression in adult erythroid cells. Transl. Res..

[59] Blumthaler M., Ambach W., Ellinger R. (1997). Increase in solar uv radiation with altitude. J. Photochem. Photobiol. B Biol..

[60] McKenzie R.L., Johnston P.V., Smale D., Bodhaine B.A., Madronich S. (2001). Altitude effects on uv spectral irradiance deduced from measurements at lauder, new zealand, and at mauna loa observatory, hawaii. J. Geophys. Res. Atmos..

[61] Rastogi R.P., Richa, Kumar A., Tyagi M.B., Sinha R.P. (2010). Molecular mechanisms of ultraviolet radiation-induced DNA damage and repair. J. Nucleic Acids.

[62] Dutta A., Yang C., Sengupta S., Mitra S., Hegde M.L. (2015). New paradigms in the repair of oxidative damage in human genome: Mechanisms ensuring repair of mutagenic base lesions during replication and involvement of accessory proteins. Cell. Mol. Life Sci..

[63] Syed A., Tainer J.A. (2018). The mre11–rad50–nbs1 complex conducts the orchestration of damage signaling and outcomes to stress in DNA replication and repair. Annu. Rev. Biochem..

[64] Muthusamy V., Piva T.J. (2010). The uv response of the skin: A review of the mapk, nfκb and tnfα signal transduction pathways. Arch. Dermatol. Res..

[65] Szoltysek K., Walaszczyk A., Janus P., Kimmel M., Widlak P. (2017). Irradiation with uv-c inhibits tnf-α-dependent activation of the nf-κb pathway in a mechanism potentially mediated by reactive oxygen species. Genes Cells.

[66] Fujita J. (1999). Cold shock response in mammalian cells. J. Mol. Microbiol. Biotechnol..

[67] Levine A.J., Oren M. (2009). The first 30 years of p53: Growing ever more complex. Nat. Rev. Cancer.

[68] Mercer T.J., Gubas A., Tooze S.A. (2018). A molecular perspective of mammalian autophagosome biogenesis. J. Biol. Chem..

[69] Hosokawa N., Sasaki T., Iemura S.-i., Natsume T., Hara T., Mizushima N. (2009). Atg101, a novel mammalian autophagy protein interacting with atg13. Autophagy.

[70] Hudmon A., Schulman H. (2002). Neuronal Ca^2+^/calmodulin-dependent protein kinase ii: The role of structure and autoregulation in cellular function. Annu. Rev. Biochem..

[71] Ross M.O., Xie Y., Owyang R.C., Ye C., Zbihley O.N., Lyu R., Wu T., Wang P., Karginova O., Olopade O.I. (2024). Ptpn2 copper-sensing relays copper level fluctuations into egfr/creb activation and associated ctr1 transcriptional repression. Nat. Commun..

[72] Bansal M., Oborn C., Danielson K., Medina D. (1989). Evidence for two selenium-binding proteins distinct from glutathione peroxidase in mouse liver. Carcinogenesis.

