# Supplementary figures and images for: Analysis of Migration and Adaptive Evolution in Tibetan Sheep Populations
Source: Animals (Basel). 2026 Jan 20;16(2):317. doi: 10.3390/ani16020317 (PMC12837694; doi:10.3390/ani16020317)

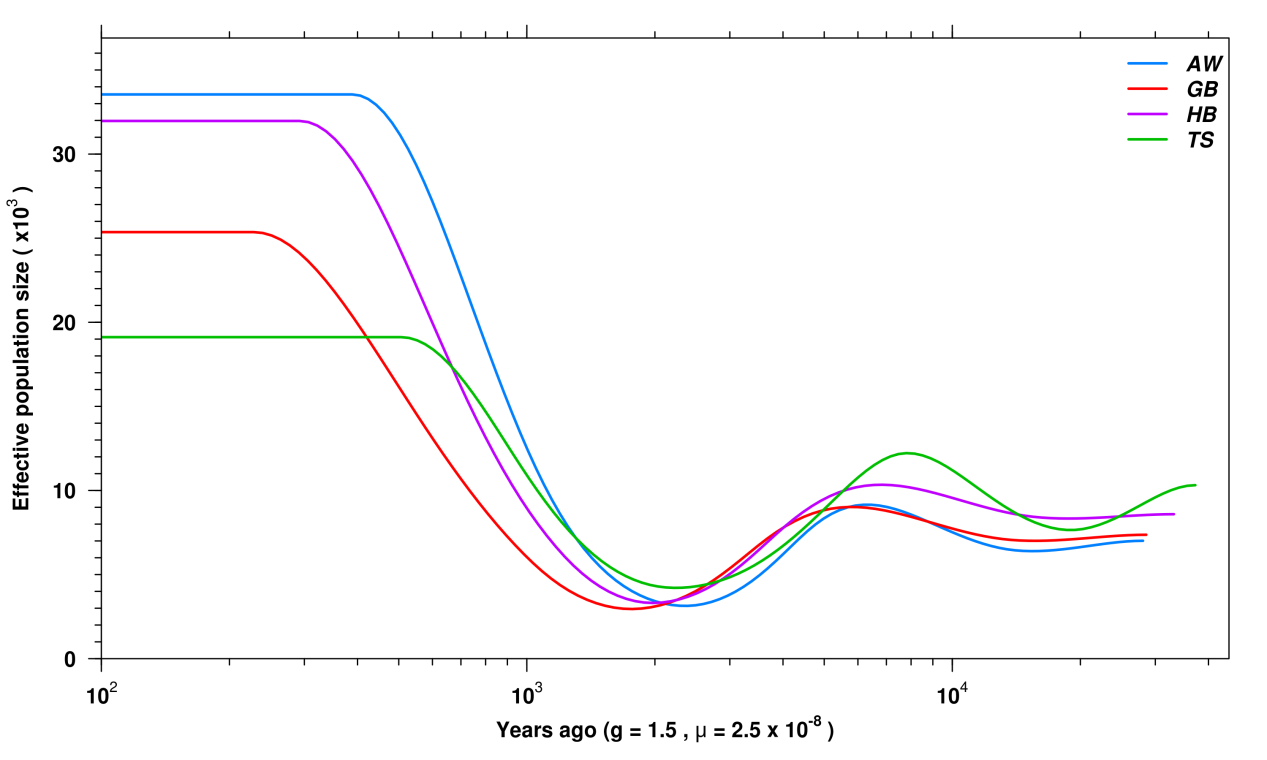

Supplement: Supplementary file 1 [file animals-16-00317-s001.zip › Supplementary Figure S1. SMCpp of four groups of analysis..png]
